# Draft Genome Sequences of Two *Lactobacillus* Strains, *L. farraginis* JCM 14108^T^ and *L. composti* JCM 14202^T^, Isolated from Compost of Distilled Shōchū Residue

**DOI:** 10.1128/genomeA.00257-14

**Published:** 2014-03-27

**Authors:** Masahiro Yuki, Kenshiro Oshima, Wataru Suda, Maki Kitahara, Keiko Kitamura, Toshiya Iida, Masahira Hattori, Moriya Ohkuma

**Affiliations:** a Biomass Research Platform Team, RIKEN Biomass Engineering Program Cooperation Division, RIKEN Center for Sustainable Resource Science, Tsukuba, Ibaragi, Japan; b Center for Omics and Bioinformatics, Graduate School of Frontier Sciences, the University of Tokyo, Kashiwa, Chiba, Japan; c Japan Collection of Microorganisms/Microbe Division, RIKEN BioResource Center, Tsukuba, Ibaragi, Japan

## Abstract

Here, we report the draft genome sequences of two type strains of *Lactobacillus*, *Lactobacillus farraginis* JCM 14108^T^ and *Lactobacillus composti* JCM 14202^T^, isolated from the compost of distilled shōchū residue. Their genome information will be useful for studies of ecological and physiological functions of these *Lactobacillus* species.

## GENOME ANNOUNCEMENT

*Lactobacillus* strains have been isolated from various environments and are frequently used as probiotics. Strains NRIC 0676 and NRIC 0689 (also available from the Japan Collection of Microorganisms as JCM 14108 and JCM 14202, respectively) were isolated from the compost of distilled shōchū (traditional Japanese spirits) residue as the type strains of the novel species *L. farraginis* and *L. composti*, respectively (1, 2). They are heterofermentative lactic acid bacteria. The 16S rRNA gene sequence analyses indicate that *L. farraginis* is closely related to *Lactobacillus hilgardii*, isolated from wine, and *Lactobacillus buchneri*, isolated from silages (1). The 16S rRNA gene sequence identities of *L. composti* to known *Lactobacillus* species are <93% (2, 3).

The genomes of *L. farraginis* JCM 14108^T^ and *L. composti* JCM 14202^T^ were sequenced using an Ion Torrent PGM system. The sequence reads, totaling 567,815 for JCM 14108^T^ and 484,069 for JCM 14202^T^, were assembled using Newbler version 2.8 (Roche) into 129 and 94 contigs, with *N*_50_ lengths of 51,335 and 87,613 bp, respectively. These assemblies resulted in draft genome sequences of 2,884,682 bp for JCM 14108^T^ and 3,449,704 bp for JCM 14202^T^, with 46.2× and 28.2× redundancies and G+C contents of 42.1 and 43.9%, respectively. A total of 3,079 and 3,863 protein-coding genes and 61 and 49 RNA-coding sequences for JCM 14108^T^ and JCM 14202^T^, respectively, were identified using the RAST server (4) and with the manual inspections detailed below.

The phosphoenolpyruvate:carbohydrate phosphotransferase (PTS) system is involved in the transport and phosphorylation of numerous carbohydrates (5, 6). *L. composti* JCM 14202^T^ has at least 17 genes related to the PTS system, whereas *L. farraginis* JCM 14108^T^ has only 6 genes. Analyses in the CAZy database (7) revealed that *L. composti* JCM 14202^T^ has genes encoding β-glucosidases belonging to the glycoside hydrolase 1 (GH1) and GH3 families, whereas *L. farraginis* JCM 14108^T^ has only those of GH3. Both strains have genes encoding α-galactosidases of GH36. *L. composti* JCM 14202^T^ has genes encoding β-galactosidases of GH2 and GH42, whereas *L. farraginis* JCM 14108^T^ has only those of GH2. In addition, *L. composti* JCM 14202^T^ has genes encoding xylanase of GH43, α-mannosidase of GH38, and α-*N*-arabinofuranosidase of GH51. Detailed analyses of the genomes of these strains will facilitate studies on the functions of *Lactobacillus* spp. in the compost of distilled shōchū residue.

### Nucleotide sequence accession numbers.

The genome sequences of *L. farraginis* JCM 14108^T^ and *L. composti* JCM 14202^T^ have been deposited at DDBJ/EMBL/GenBank under accession no. BAKI01000001 to BAKI01000129 and BAMK01000001 to BAMK01000094, respectively.
